# Supplementary material for: Gut Bacteriomes and Ecological Niche Divergence: An Example of Two Cryptic Gastropod Species
Source: Biology (Basel). 2023 Dec 13;12(12):1521. doi: 10.3390/biology12121521 (PMC10740740; doi:10.3390/biology12121521)
Supplement: Supplementary file 1 [file biology-12-01521-s001.zip › supplement S1.pdf]

Gut bacteriomes and Ecological Niche Divergence: An Example of Two Cryptic Gastropod Species

Elizaveta Gafarova [1,*], Dmitrii Kuracji [1], Karina Sogomonyan [2], Ivan Gorokhov [1], Dmitrii Polev [3], Ekaterina Zubova [1], Elena Golikova [1], Andrey Granovitch [1] and Arina Maltseva [1]

[1] Department of Invertebrate Zoology, St. Petersburg State University, 199034 St. Petersburg, Russia; dkuracij@gmail.com (D.K.); zubova.15@list.ru (E.Z.); a.granovich@spbu.ru (A.G.)
[2] Center for Bioinformatics and Algorithmic Biotechnology, St. Petersburg State University, 199034 St. Petersburg, Russia; karinasog96@gmail.com
[3] Department of Epidemiology, St. Petersburg Pasteur Institute, Mira Street 14, 197101 St. Petersburg, Russia; brantoza@gmail.com
* Correspondence: st047483@student.spbu.ru or orhidea-palma@yandex.ru

ABSTRACT

Symbiotic microorganisms may provide their hosts with abilities critical to their occupation of microhabitats. Gut (intestinal) bacterial communities aid animals to digest substrates that are either innutritious or toxic, as well as support their development and physiology. The role of microbial communities associated with sibling species in the hosts' adaptation remains largely unexplored. In this study, we examined the composition and plasticity of the bacteriomes in two sibling intertidal gastropod species, *Littorina fabalis* and *L. obtusata*, which are sympatric but differ in microhabitats. We applied 16S rRNA gene metabarcoding and shotgun sequencing to describe associated microbial communities and their spatial and temporal variation. A significant drop in the intestinal bacteriome diversity was revealed during the cold season, which may reflect temperature-related metabolic shifts and changes in snail behavior. Importantly, there were significant interspecies differences in the gut bacteriome composition in summer but not in autumn. The genera *Vibrio*, *Aliivibrio*, *Moritella* and *Planktotalea* were found to be predominantly associated with *L. fabalis*, while *Granulosicoccus*, *Octadecabacter*, *Colwellia*, *Pseudomonas*, *Pseudoalteromonas* and *Maribacter* were found to be mostly associated with *L. obtusata*. Based on these preferential associations, we analyzed the metabolic pathways' enrichment. We hypothesized that the *L. obtusata* gut bacteriome contributes to decomposing algae and detoxifying polyphenols produced by fucoids. Thus, differences in the sets of associated bacteria may equip their closely phylogenetically related hosts with a unique ability to occupy specific micro-niches.

Citation: Gafarova, E.; Kuracji, D.; Sogomonyan, K.; Gorokhov, I.; Polev, D.; Zubova, E.; Golikova, E.; Granovitch, A.; Maltseva, A. Gut bacteriomes and Ecological Niche Divergence: An Example of Two Cryptic Gastropod Species. *Biology* **2023**, *12*, 1521. https://doi.org/10.3390/12121521

Academic Editor: Richard F. Lee

Received: 26 October 2023
Revised: 4 December 2023
Accepted: 8 December 2023
Published: 13 December 2023

Copyright: © 2023 by the authors. Submitted for possible open access publication under the terms and conditions of the Creative Commons Attribution (CC BY) license (https://creativecommons.org/licenses/by/4.0/).

Supplement

a)

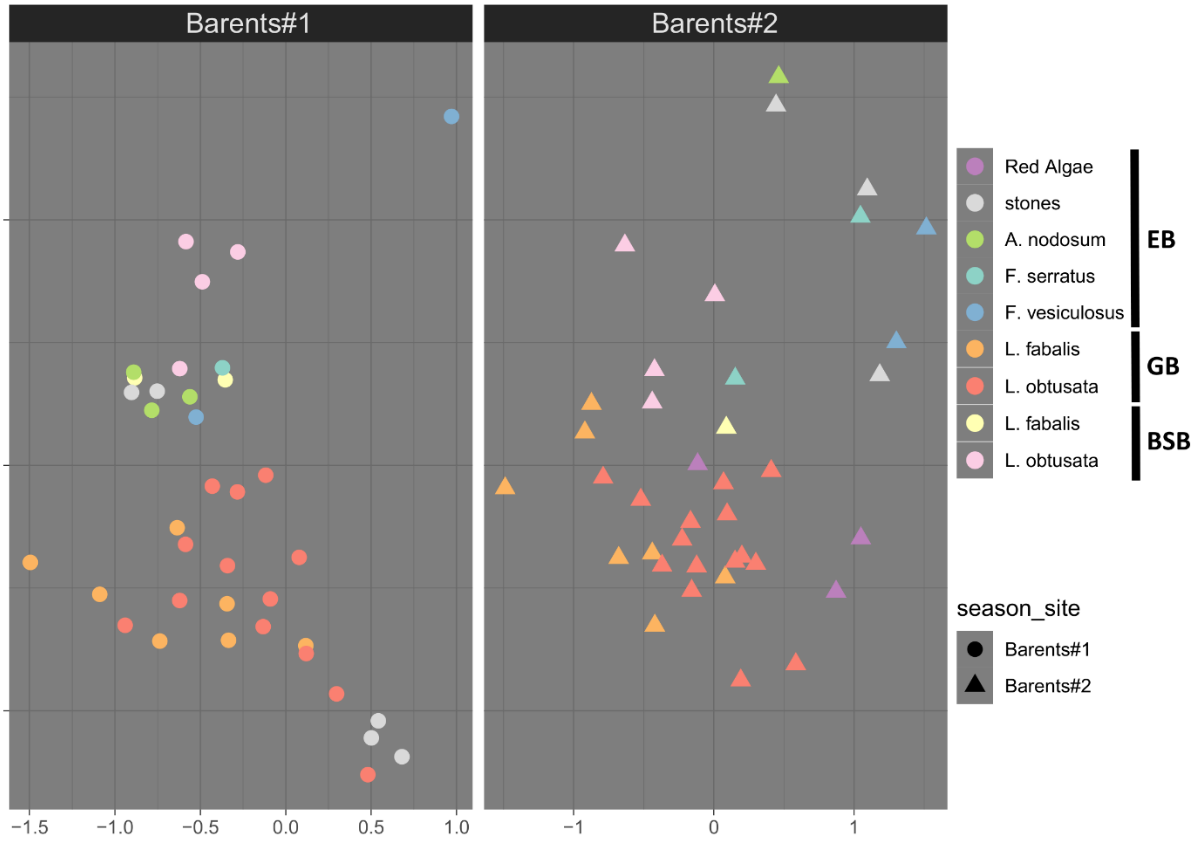

b)

```
             Df      SS      MS     Rsq      F       Z Pr(>F)
site          1  0.4584 0.45836 0.04141 1.8019 1.56944  0.063 .
species       1  0.6297 0.62969 0.05689 2.4754 2.34604  0.012 *
site:species  1  0.3137 0.31373 0.02835 1.2333 0.70154  0.238
Residuals    38  9.6665 0.25438 0.87335
Total        41 11.0683
---
Signif. codes:  0 ‘***’ 0.001 ‘**’ 0.01 ‘*’ 0.05 ‘.’ 0.1 ‘ ’ 1

Call: lm.rrpp(f1 = rev_full_guts_barents_2_bray_snails ~ site * species,      data = rev_full_guts_barents_2_df)
```

Figure S1. Comparison of the Barents Sea bacteriomes**. a) nMDS of the whole bacteriome dataset, only samples from the Barents Sea are represented in facets.** Colours mark different types of samples, shapes stand for sites. EB – environmental bacteriome, GB – gut bacteriome, BSB – body surface bacteriome. Barents#1 – Oscar Bay, the Barents Sea; Barents#2 – Yarnyshnaya Bay, the Barents Sea. **b) Permutational analysis of variance results.** The two-factor test was performed on subsampled gut dataset (p-value for the “site” factor is 0.06; p-value for the “host species” factor is 0.012; p-value for the factors interaction is 0.238).

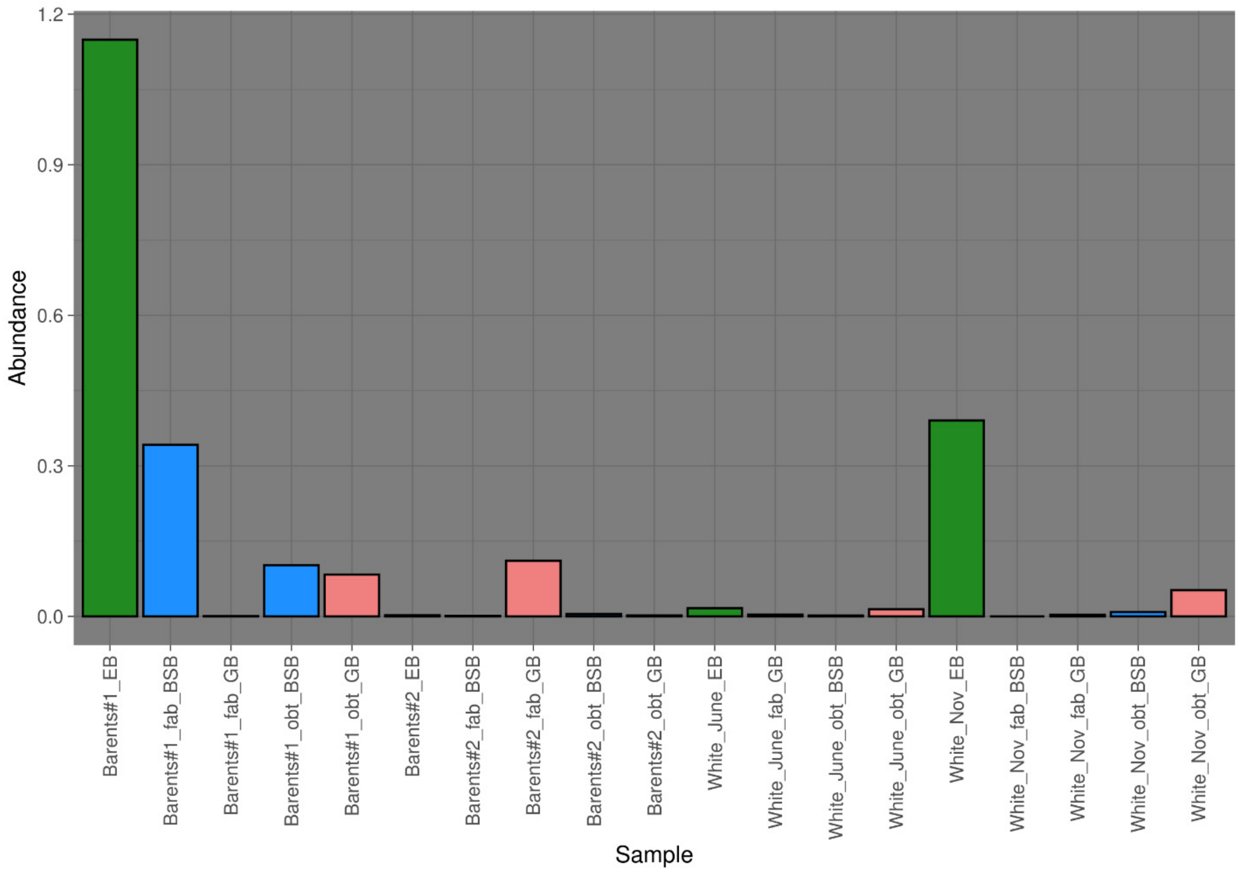

Figure S2. Relative abundance of the *Acinetobacter* sp. in all analyzed samples. Colours mark sample type. Green – EB, environmental bacteriome; Blue – BSB, body surface bacteriome; Red – GB, gut bacteriome. Barents#1 – Oscar Bay, the Barents Sea; Barents#2 – Yarnyshnaya Bay, the Barents Sea; White_June – the summer (June 2022) samples from the White Sea; White_Nov – the autumn (November 2021) samples from the White Sea; obt – *Littorina obtusata*; fab – *L. fabalis*.

a)

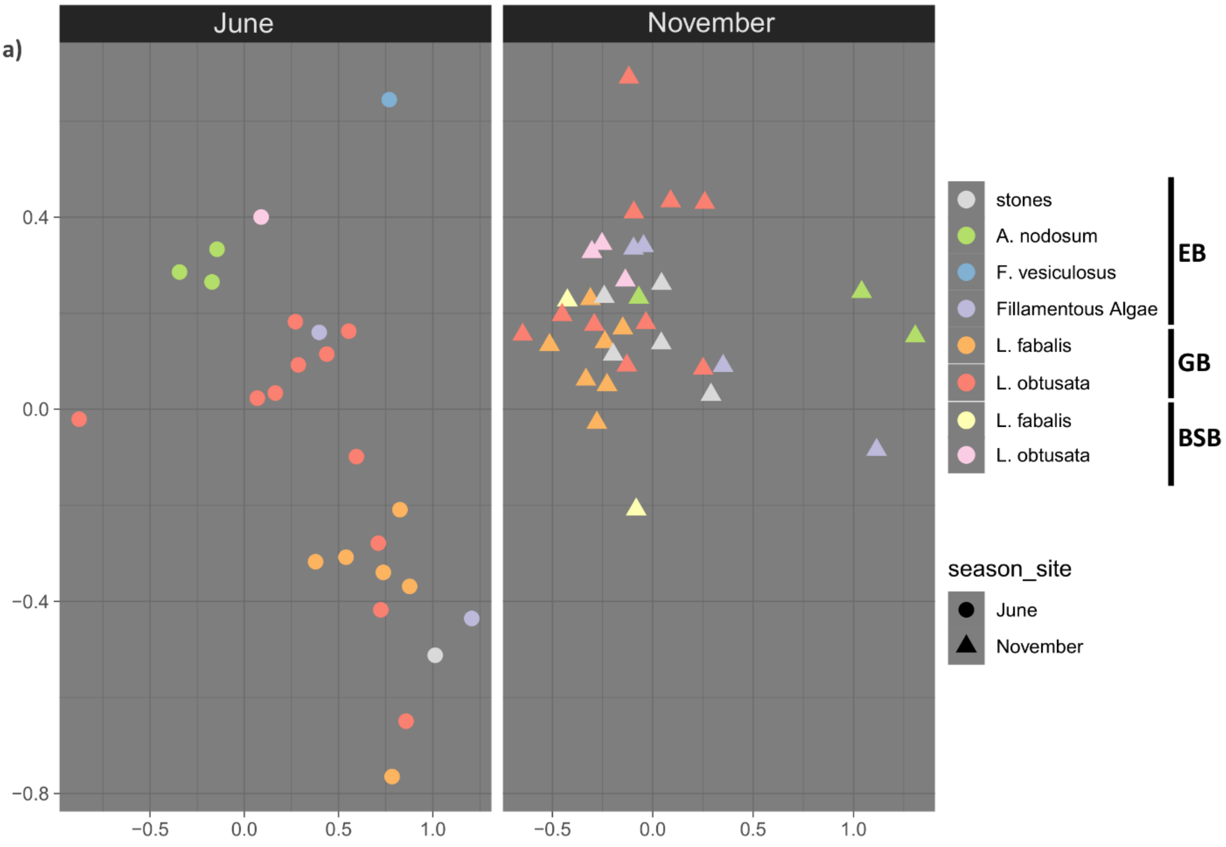

b)

June 2022

```
          Df      SS      MS     Rsq      F      Z Pr(>F)
species    1 0.35046 0.35046 0.11204 1.8927 1.6128  0.048 *
Residuals 15 2.77754 0.18517 0.88796
Total     16 3.12800
---
Signif. codes:  0 ‘***’ 0.001 ‘**’ 0.01 ‘*’ 0.05 ‘.’ 0.1 ‘ ’ 1

Call: lm.rrpp(f1 = june2022_rev__white_bray_snails ~ species, iter = 999,      data = june2022_rev__white_df)
```

November 2021

```
          Df      SS       MS    Rsq      F        Z Pr(>F)
species    1 0.03493 0.034925 0.0544 0.8629 -0.20929  0.568
Residuals 15 0.60712 0.040475 0.9456
Total     16 0.64204

Call: lm.rrpp(f1 = nov2021_rev__white_bray_snails ~ species, iter = 999,      data = nov2021_rev__white_df)
```

Figure S3. Comparison of the White Sea bacteriomes. **a) nMDS of the whole bacteriome dataset, only samples from the White Sea are represented in facets.** Colours mark different types of samples, shapes stand for sites. EB – environmental bacteriome, GB – gut bacteriome, BSB – body surface bacteriome. White_June – the summer (June 2022) samples from the White Sea; White_Nov – the autumn (November 2021) samples from the White Sea **b) Permutational analysis of variance results.** The tests were performed on subsampled gut dataset. The test on summer data (June 2022) showed significant differences between species (p-value = 0.048). The test on autumn data (November 2021) did not show significant differences between species (p-value = 0.568).

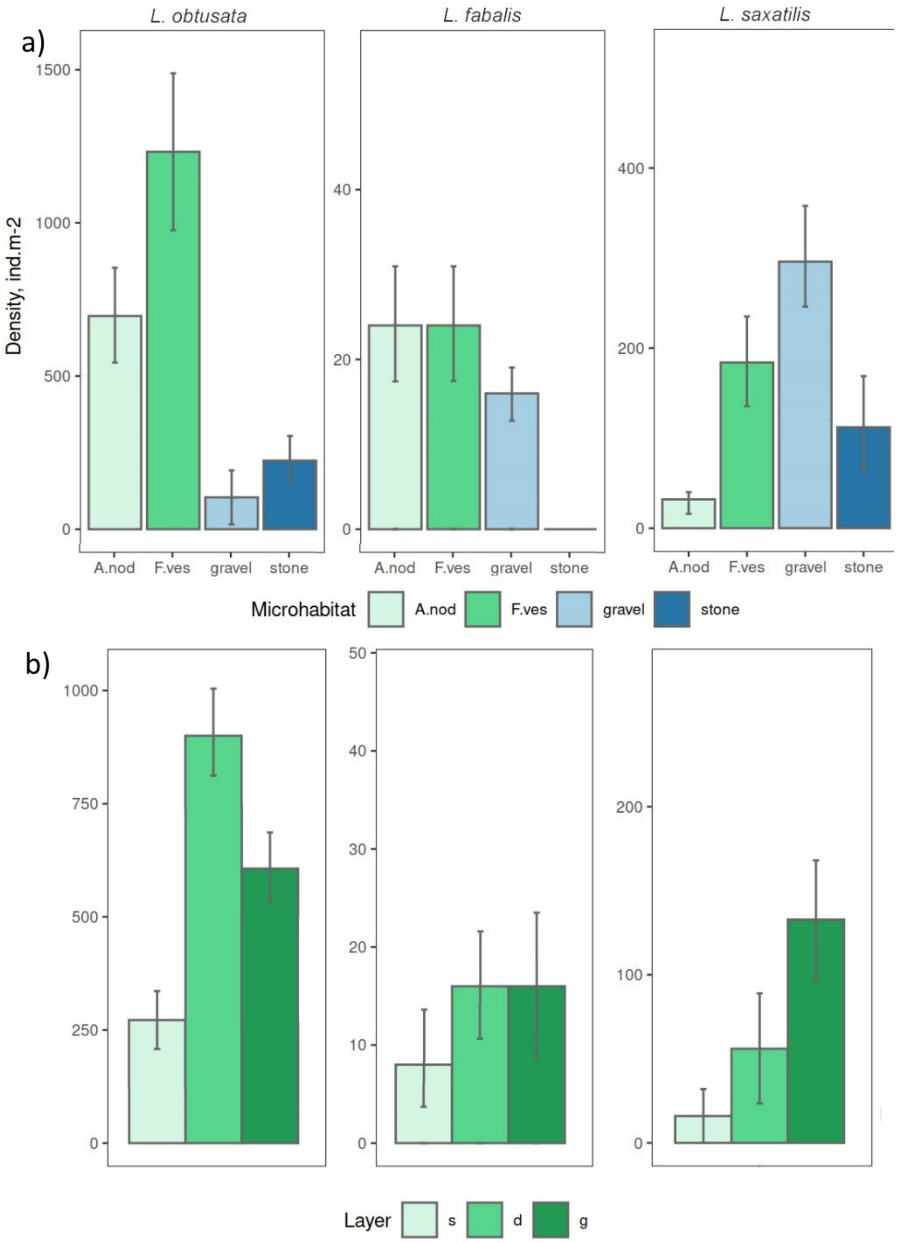

Figure S4. *Littorina* microniche distribution. **a) Microhabitat distribution of several Littorina species.** A.nod – *Ascophyllum nodosum*-associated settlements; F.ves – *Fucus vesiculosus*-associated settlements; gravel – gravel-associated settlements; stone – stone-associated settlements. **b) Layer-specific distribution of several *Littorina* species in fucoid canopy.** s – surface, d – depth, g – gravel. The distribution was assessed following the specific sample collection design. Snails from the open gravel and the stone surface were collected directly from quadrats (0.04 m^2, 0.20 m × 0.20 m). Sampling on fucoids was performed in layers: the snails were collected separately from the surface of the kelps, from the "depth" of the fucoid canopy, and from the gravel substrate below. This approach made it possible to calculate the distribution of individuals in different fucoid layers [42].

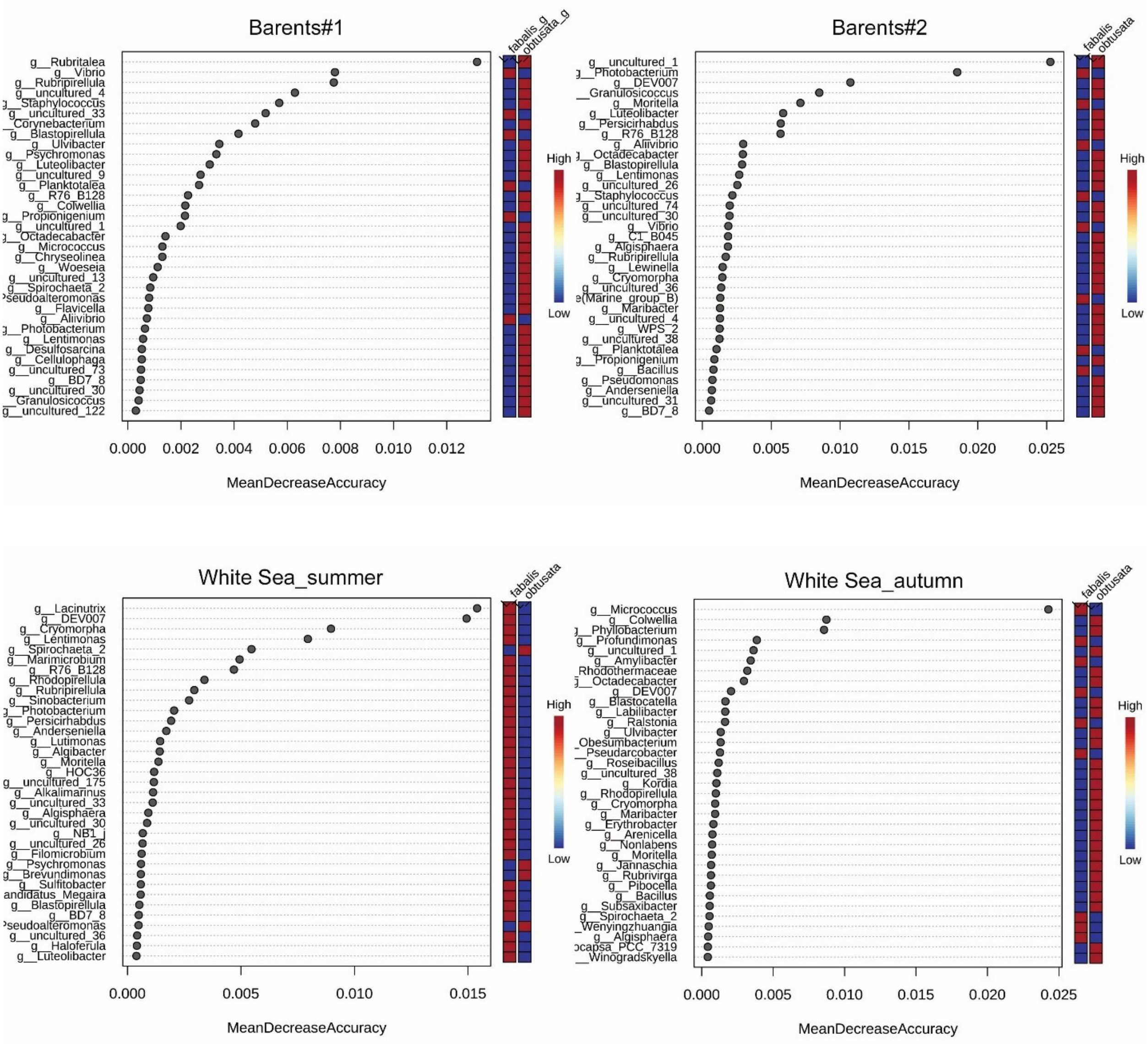

Figure S5. The Random Forest analysis results. Genera specific to the particular host species (*Littorina obtusata* or *L. fabalis*) gut bacteriome are shown in red. The higher value of mean decrease accuracy indicates the importance of the genus in a microbiome. Barents#1 – Oscar Bay, the Barents Sea; Barents#2 – Yarnyshnaya Bay, the Barents Sea; White_summer – the summer (June 2022) samples from the White Sea; White_autumn – the autumn (November 2021) samples from the White Sea.

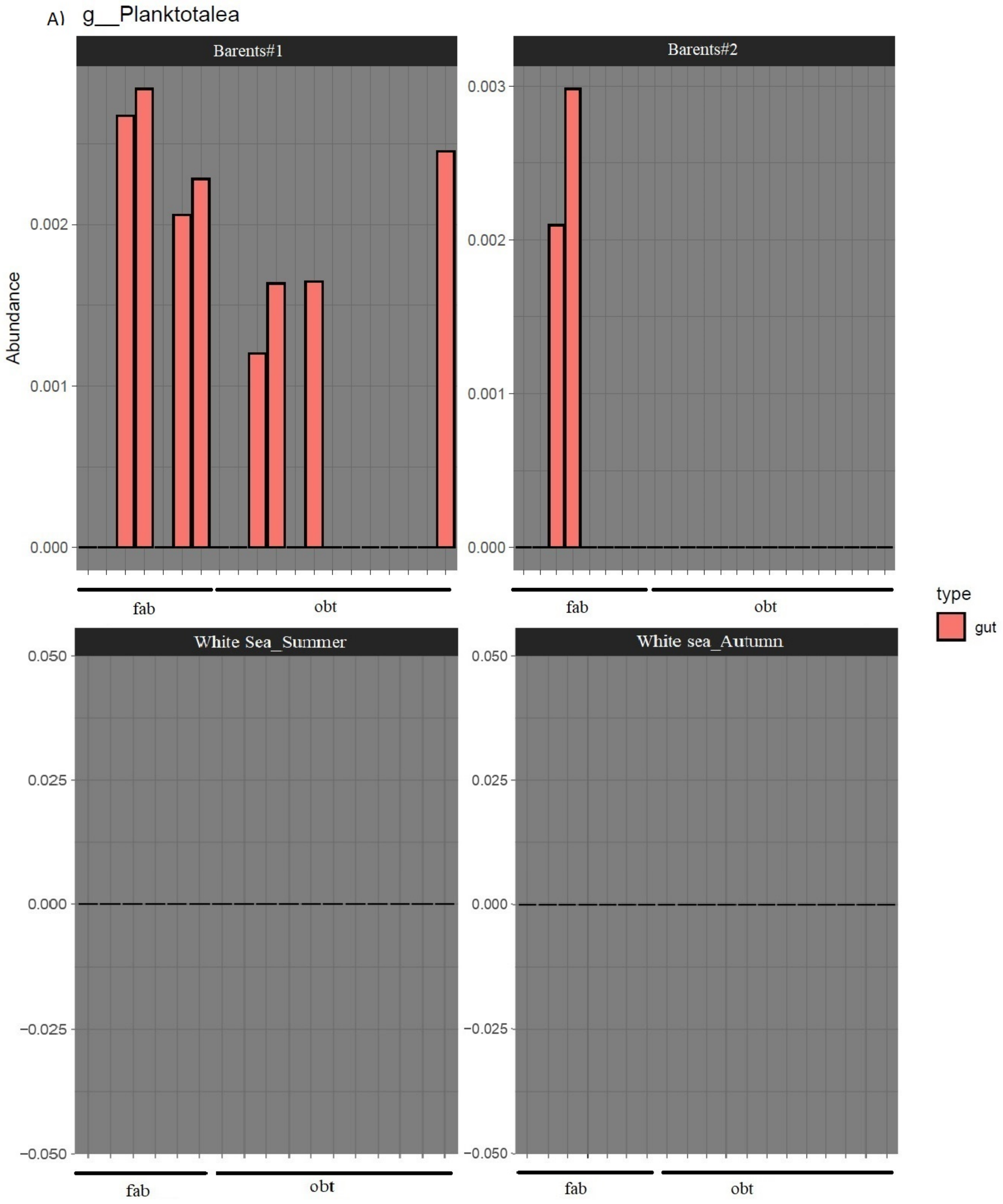

A) g__Planktotalea
Barents#1
Barents#2
White Sea_Summer
White sea_Autumn
Abundance
0.003
0.002
0.001
0.000
0.050
0.025
−0.025
−0.050
fab
obt
type
gut

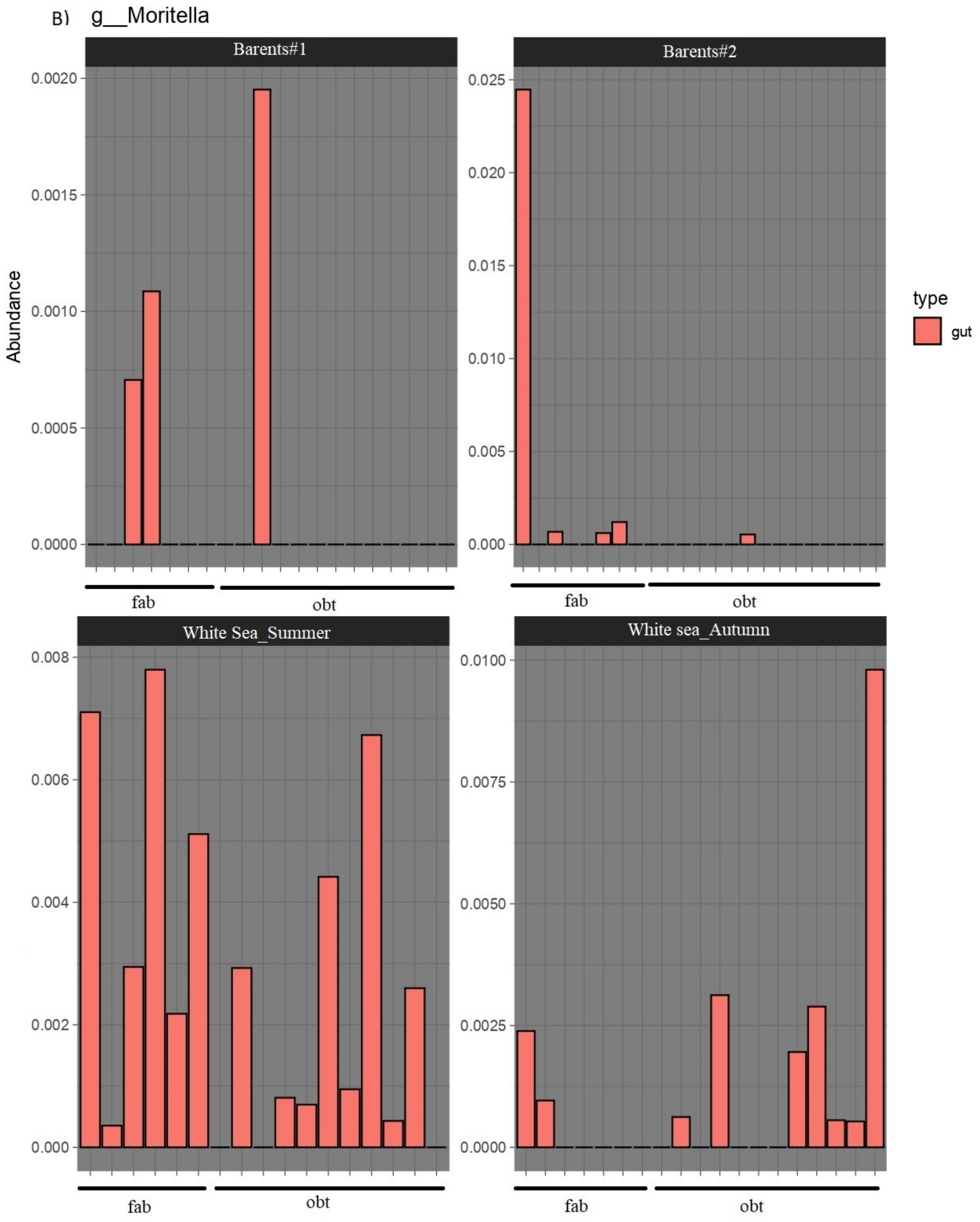
B)
g__Moritella
Barents#1
Barents#2
White Sea_Summer
White sea_Autumn
Abundance
0.0020
0.0015
0.0010
0.0005
0.0000
0.025
0.020
0.015
0.010
0.005
0.000
0.008
0.006
0.004
0.002
0.000
0.0100
0.0075
0.0050
0.0025
0.0000
fab
obt
type
gut

c) g__Aliivibrio

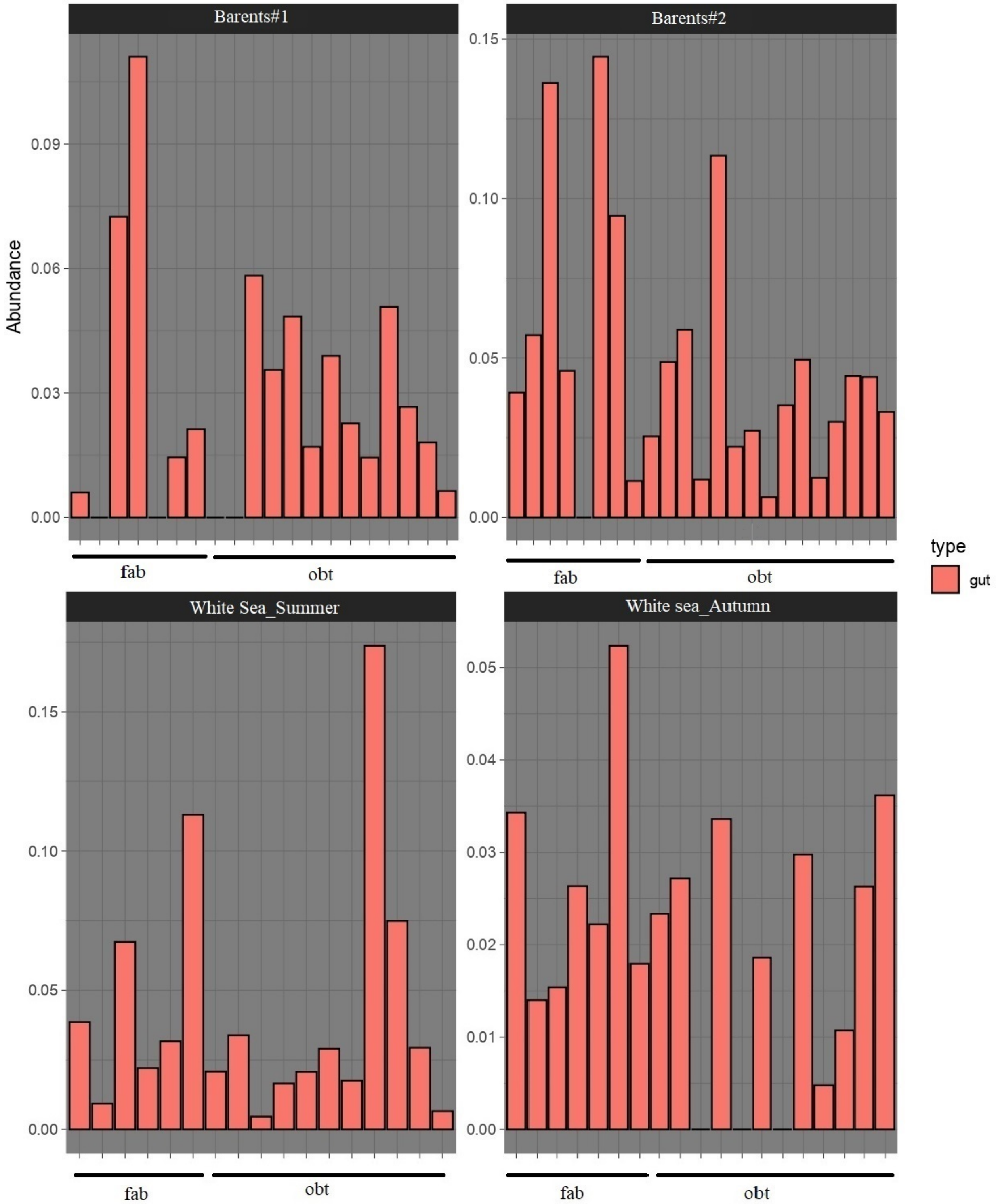

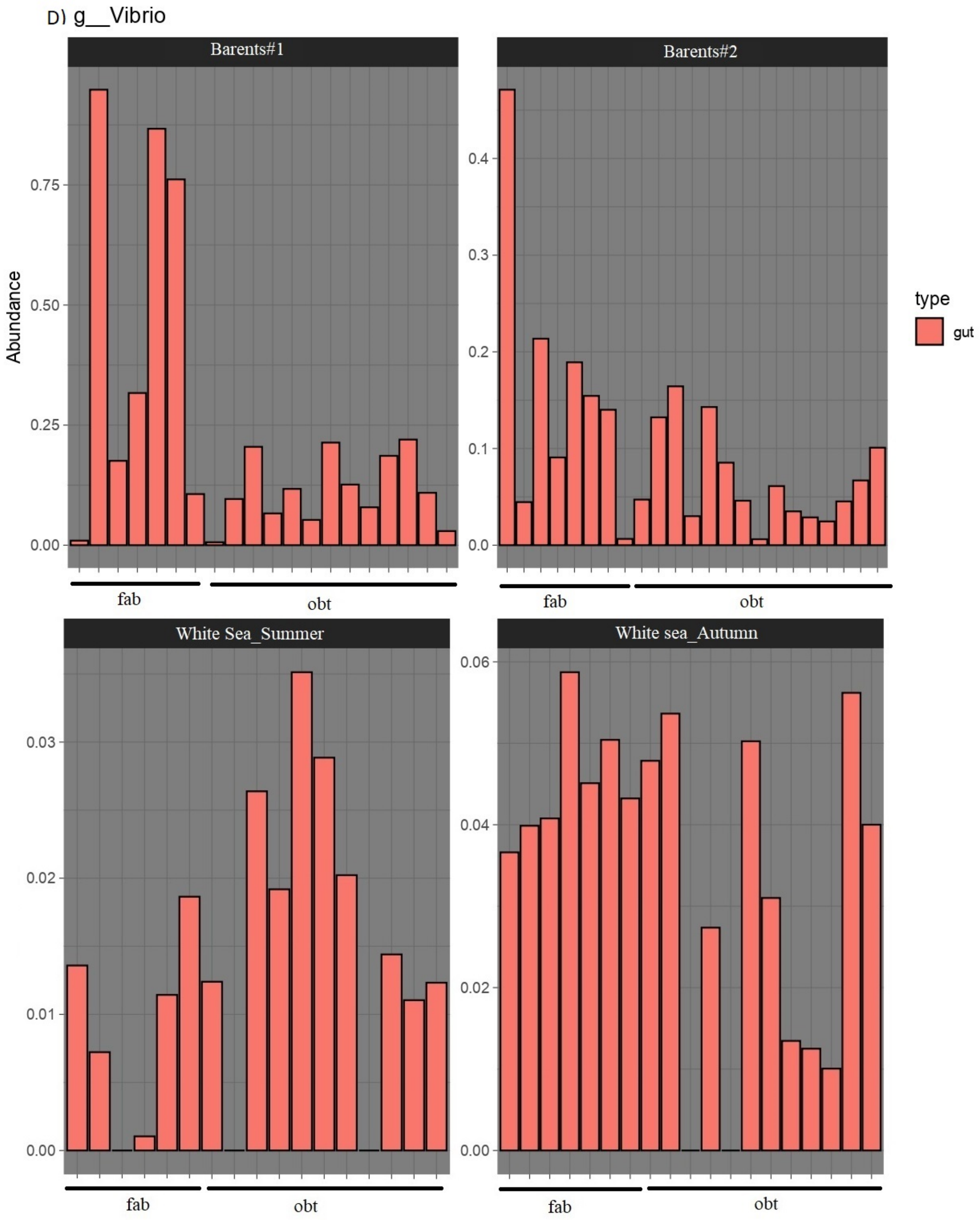

Figure S6. Relative abundance of the bacteria predominantly abundant in *Littorina fabalis* gut samples. Distributions of relative abundance values of (A) *Planktotalea* sp., (B) *Moritella* sp., (C) *Aliivibrio* sp., and (D) *Vibrio* sp. among the gut species.

Barents#1 – Oscar Bay, the Barents Sea; Barents#2 – Yarnyshnaya Bay, the Barents Sea; White_Summer – the summer (June 2022) samples from the White Sea; White_Autumn – the autumn (November 2021) samples from the White Sea.

A) g__Maribacter

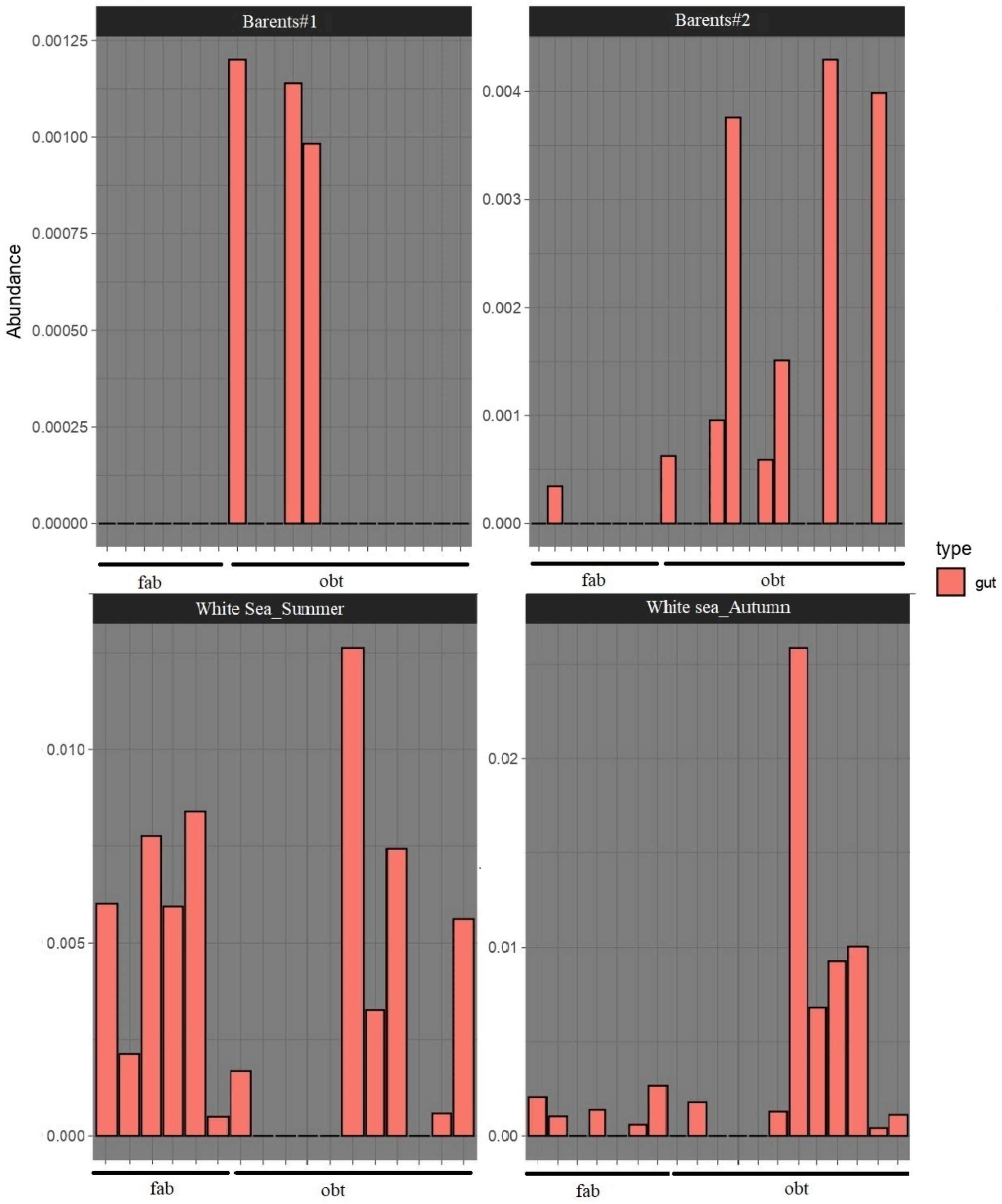

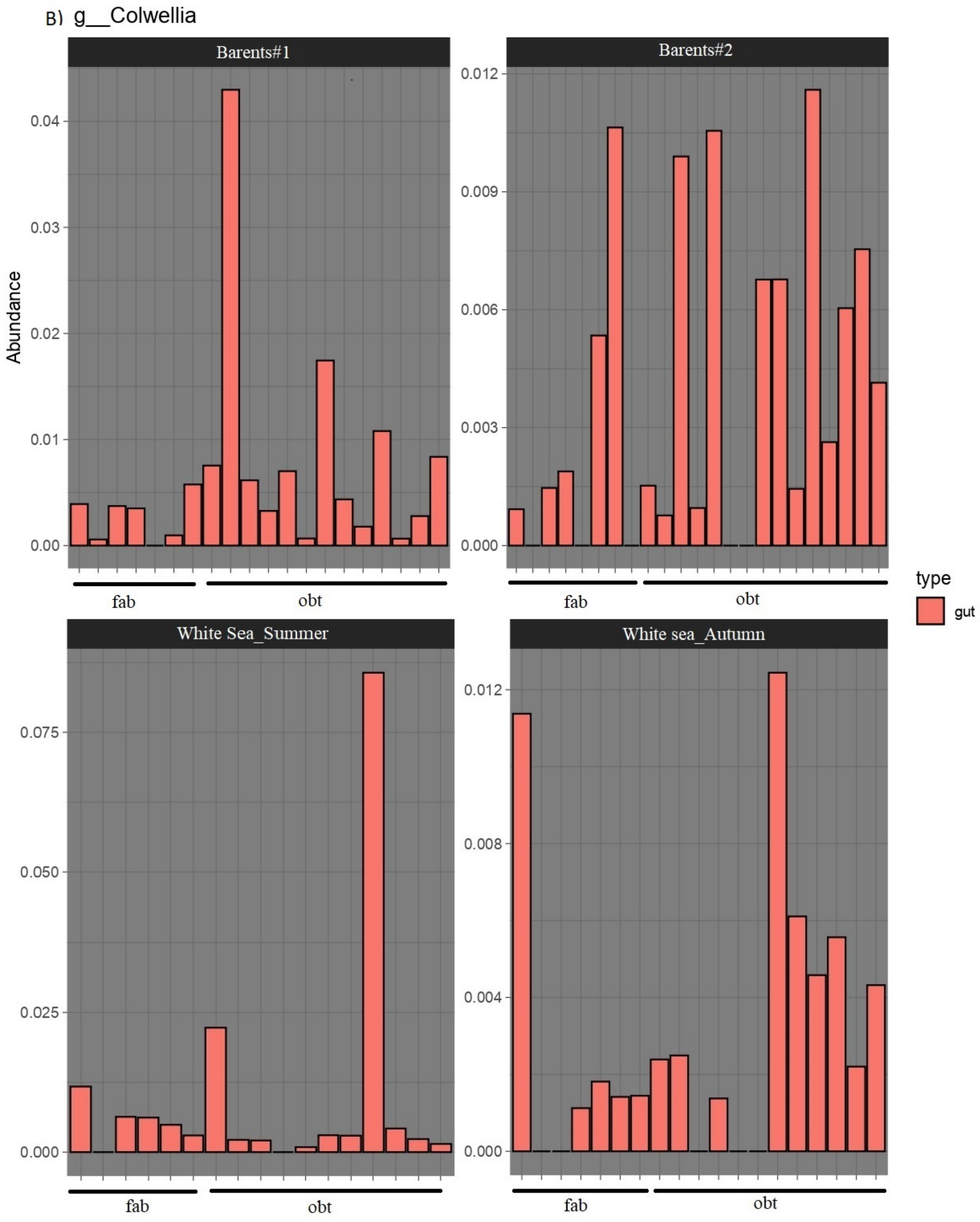
B) g__Colwellia
Barents#1
Barents#2
White Sea_Summer
White sea_Autumn
Abundance
0.04
0.03
0.02
0.01
0.00
0.012
0.009
0.006
0.003
0.000
0.075
0.050
0.025
0.000
0.008
0.004
fab
obt
type
gut

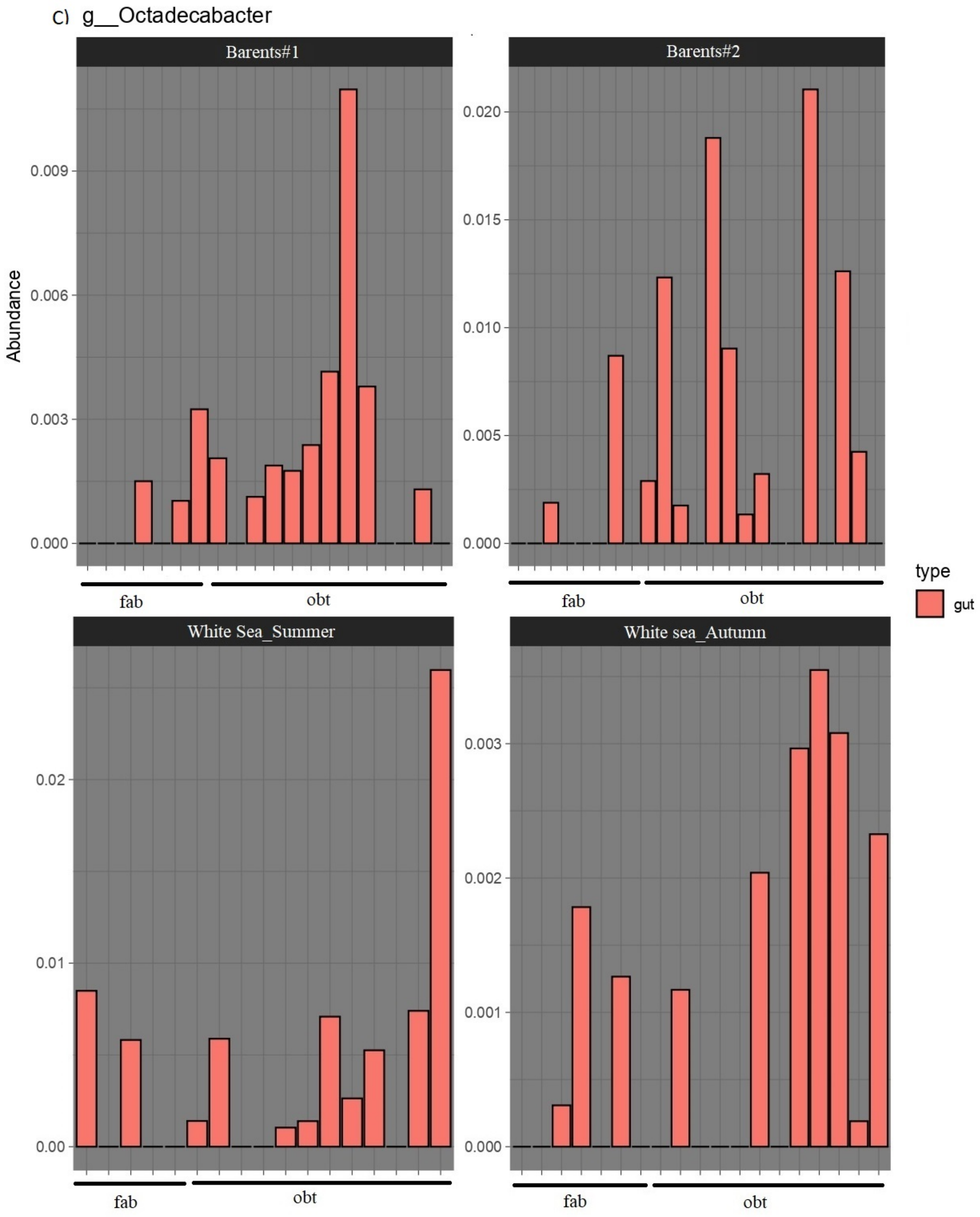
c) g__Octadecabacter
Barents#1
Barents#2
White Sea_Summer
White sea_Autumn
Abundance
0.000
0.003
0.006
0.009
0.005
0.010
0.015
0.020
0.00
0.01
0.02
0.001
0.002
0.003
fab
obt
type
gut

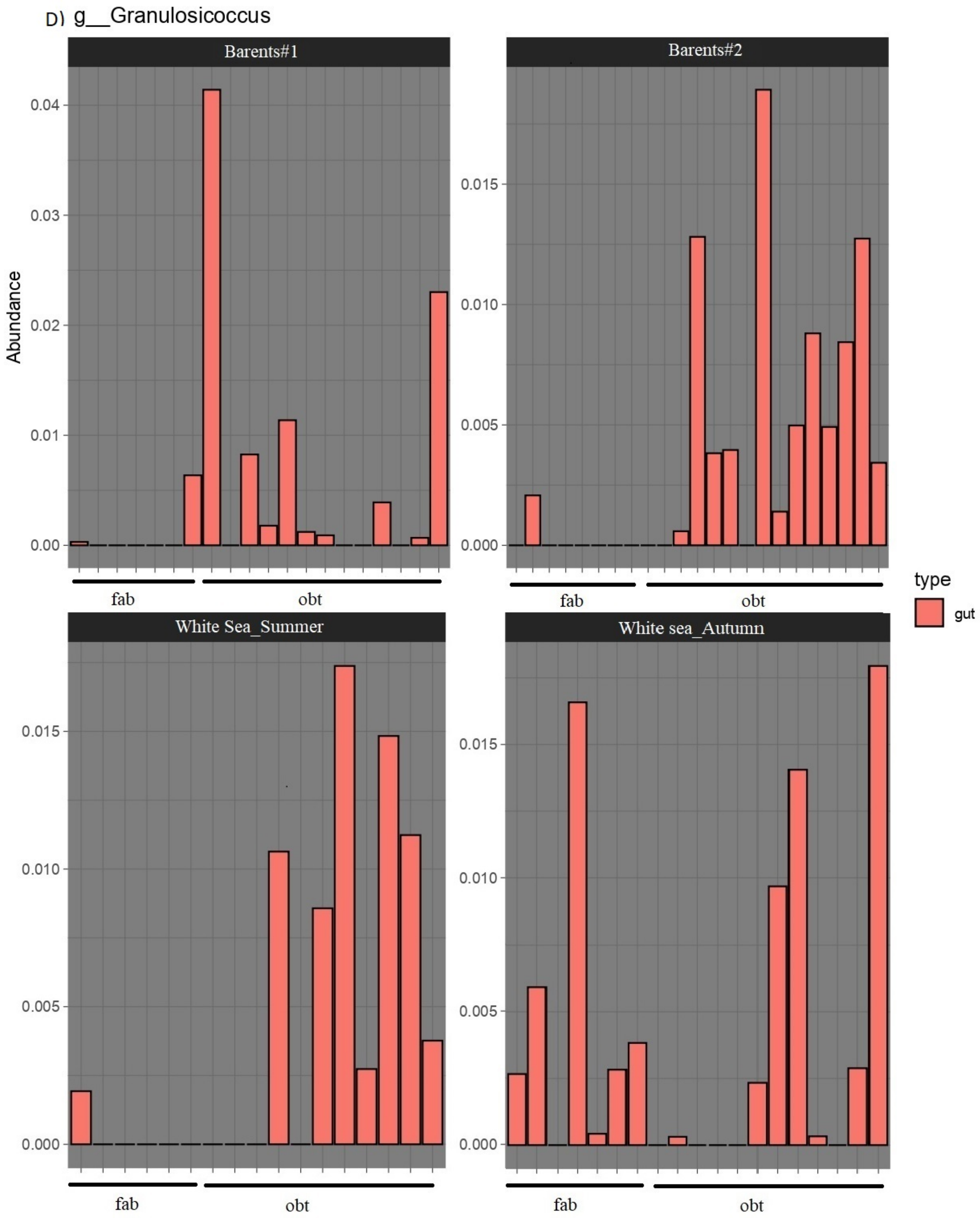
D) g__Granulosicoccus
Barents#1
Barents#2
White Sea_Summer
White sea_Autumn
Abundance
0.04
0.03
0.02
0.01
0.00
0.015
0.010
0.005
0.000
fab
obt
type
gut

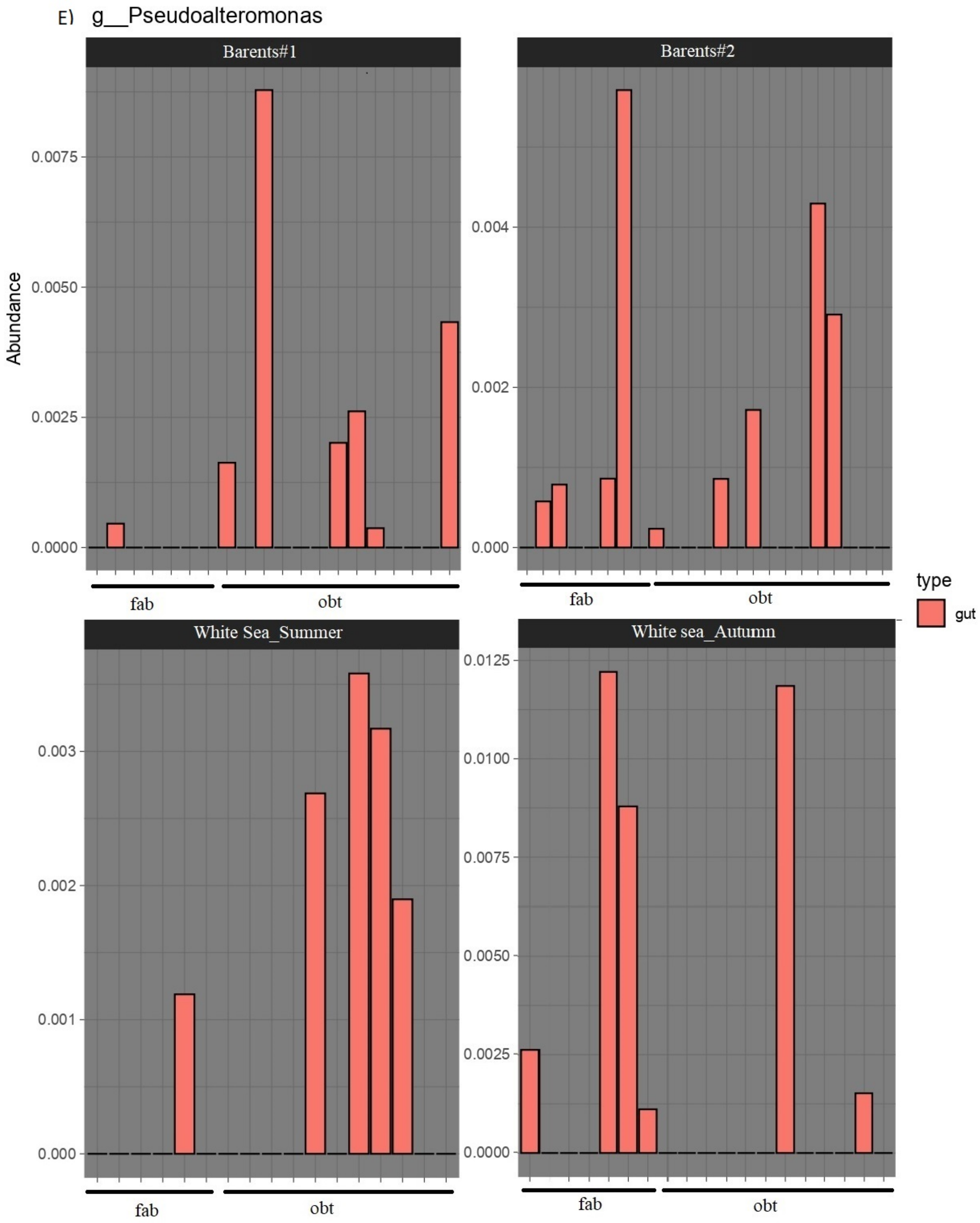

E)
g__Pseudoalteromonas
Barents#1
Barents#2
White Sea_Summer
White sea_Autumn
Abundance
0.0075
0.0050
0.0025
0.0000
0.004
0.002
0.000
0.003
0.002
0.001
0.000
0.0125
0.0100
0.0075
0.0050
0.0025
0.0000
fab
obt
type
gut

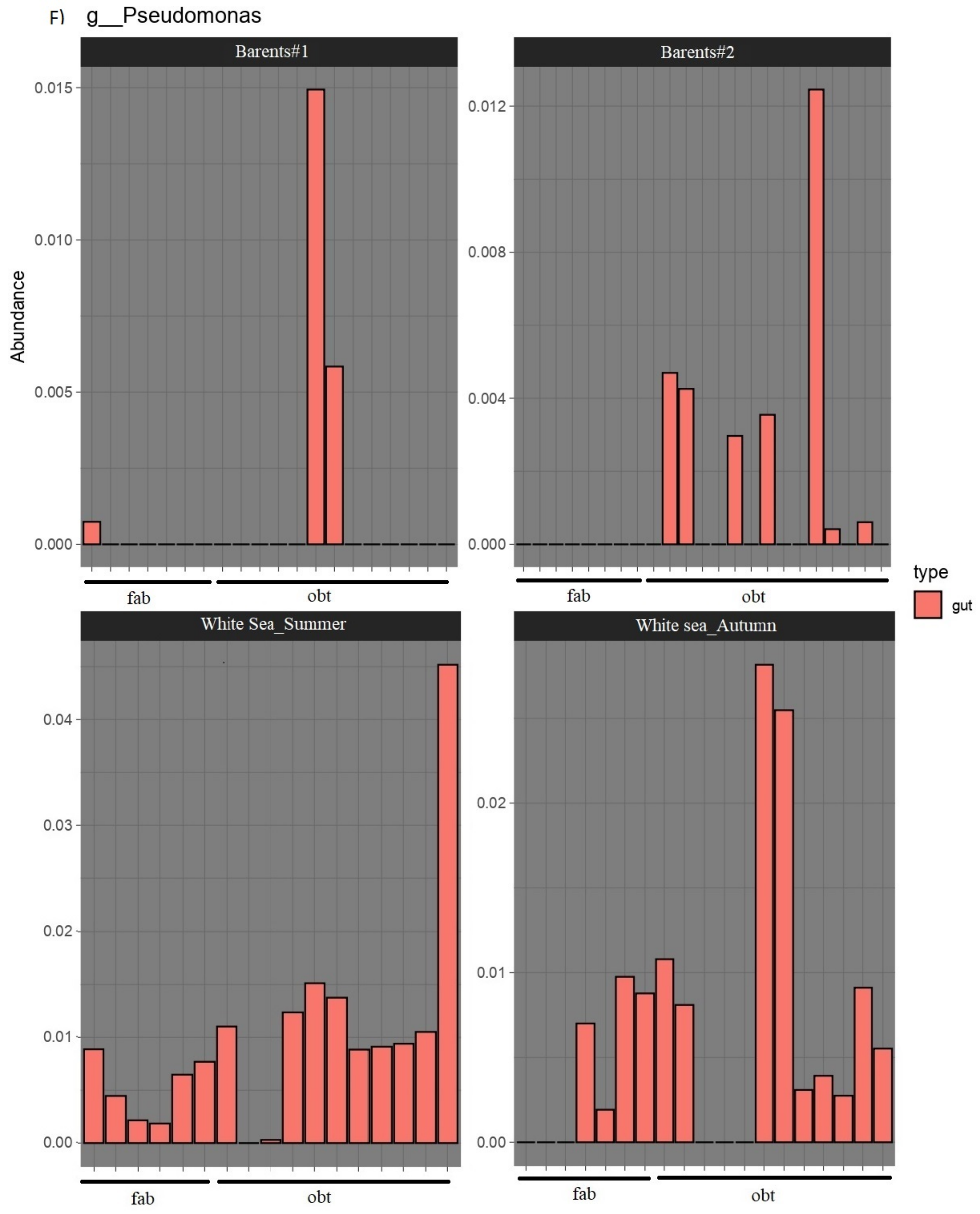

Figure S7. Relative abundance of the bacteria predominantly abundant in *Littorina obtusata*. *fabalis* gut samples. Distributions of relative abundance values of (A) *Maribacter* sp., (B) *Colwellia* sp., (C) *Octadecabacter* sp., (D) *Granulosicoccus* sp., (E) *Pseudoalteromonas* sp., and (F) *Pseudomonas* sp. among the gut species.

Barents#1 – Oscar Bay, the Barents Sea; Barents#2 – Yarnyshnaya Bay, the Barents Sea; White_Summer – the summer (June 2022) samples from the White Sea; White_Autumn – the autumn (November 2021) samples from the White Sea.
